# PPI‐Induced Changes in Plasma Metabolite Levels Influence Total Hip Bone Mineral Density in a UK Cohort

**DOI:** 10.1002/jbmr.4754

**Published:** 2022-12-30

**Authors:** Xinyuan Zhang, Adewale S. Adebayo, Dongmeng Wang, Yasrab Raza, Max Tomlinson, Hannah Dooley, Ruth C.E. Bowyer, Kerrin S. Small, Claire J. Steves, Tim D. Spector, Emma L. Duncan, Alessia Visconti, Mario Falchi

**Affiliations:** ^1^ Department of Twins Research & Genetics Epidemiology King's College London London UK; ^2^ Present address: NIHR Leicester Biomedical Research Centre, Department of Cardiovascular Sciences University of Leicester Leicester UK

**Keywords:** PROTON PUMP INHIBITORS, BONE MINERAL DENSITY, GUT MICROBIOME, GUT METABOLOME, HOST METABOLOME

## Abstract

Proton pump inhibitors (PPIs) are among the most used drugs in the UK. PPI use has been associated with decreased bone mineral density (BMD) and increased fracture risk, although these results have been inconsistent. We hypothesized that PPI could modulate BMD by altering gut and/or host systemic metabolic environments. Using data from more than 5000 British male and female individuals, we confirmed that PPI use is associated with decreased lumbar spine and total hip BMD. This effect was not mediated through the gut microbiome. We suggest here that PPI use may influence total hip BMD, both directly and indirectly, via plasma metabolites involved in the sex hormone pathway. © 2022 The Authors. *Journal of Bone and Mineral Research* published by Wiley Periodicals LLC on behalf of American Society for Bone and Mineral Research (ASBMR).

## Introduction

Proton pump inhibitors (PPIs) are among the most commonly used drugs in the UK,^(^ ^1^ ^)^ prescribed for peptic ulcer and gastroesophageal reflux disease or co‐prescribed to prevent adverse effects from drugs such as nonsteroidal anti‐inflammatory drugs. PPIs antagonize gastric hydrogen‐potassium ATPase through covalent binding, blocking the secretion of H^+^ ions into the gastric lumen, and thereby inhibiting gastric acidity.

Gastric acids are important for the absorption of diverse nutrient groups such as vitamins, iron, and calcium. For example, the acidic environment in the stomach helps convert soluble calcium salts to ionized soluble calcium, facilitating calcium absorption in the intestine. Several studies have suggested that this PPI‐induced lowered intestinal calcium absorption may cause lower bone mineral density (BMD) and increased vertebral and non‐spinal fracture rates.^(^ ^2^, ^3^ ^)^ In 2010, the US Food and Drug Administration added increased risk of hip, wrist, and vertebral fractures as possible side effects of PPIs, after these were reported by several epidemiological studies.^(^ ^4^, ^5^, ^6^ ^)^ However, these observations are still controversial, as other studies have not found any association between long‐term PPI use and loss of BMD and/or fracture risk.^(^ ^7^, ^8^, ^9^ ^)^

Several potential mechanisms by which PPI use might affect BMD have been suggested, including the already‐mentioned PPI‐induced reduced calcium absorption, leading to secondary hyperparathyroidism.^(^ ^10^ ^)^ PPI may also reduce magnesium absorption.^(^ ^11^ ^)^ PPI might also act directly on bone cells, with in vitro evidence that incubation of omeprazole with fish scales directly upregulates osteoclastic markers in the cells.^(^ ^12^ ^)^ A recent in vitro study using the mouse osteoblast cell line MC3T3‐E1 showed that lansoprazole could lead to intracellular calcium overload, inducing endoplasmic reticulum stress and causing osteoblast apoptosis; moreover, 6‐month treatment with lansoprazole in mouse models significantly decreased femoral BMD, in a dose‐dependent manner.^(^ ^13^ ^)^

Metabolomics is a promising agnostic tool to better understand metabolic changes induced by PPI use, and for identifying molecular biomarkers of PPI‐mediated BMD loss. Several animal and human studies have been carried out to identify metabolites associated with low BMD and osteoporosis.^(^ ^14^, ^15^, ^16^ ^)^ Candidate metabolites belong to different categories, including lipids and amino acids, and the underlying causal molecular mechanisms of these findings are unclear. A recent study identified 27 plasma metabolites associated with lumbar spine and femoral neck BMD and incorporating these metabolites into a prediction model improved fracture prediction beyond conventional risk factors.^(^ ^14^ ^)^ However, no studies have yet assessed whether changes in the metabolic environment induced by PPI use are involved in modulating BMD.

PPI use can also affect the composition of the gut microbiome. Physiologically, the highly acidic gastric environment functions as a barrier to prevent bacteria from entering the intestine. The low acidity environment induced by PPIs may increase the risk of enteric infections such as *Clostridium difficile* infection.^(^ ^17^ ^)^ Cross‐sectional studies have observed overrepresentations in the gut of oral bacteria genera such as *Streptococcus* and *Rothia*, increased abundance of the bacteria families *Micrococcaceae* and *Lactobacillaceae*, and decreased abundance of the families *Lachnospiraceae* and *Ruminococcaceae*.^(^ ^18^, ^19^, ^20^, ^21^ ^)^ Increased *Streptococcaceae* or *Streptococcus* abundances have been reported by multiple studies, in both healthy individuals^(^ ^22^, ^23^ ^)^ and individuals with cirrhosis^(^ ^24^, ^25^ ^)^ who were on PPI. Interestingly, the gut microbiome has been suggested to influence bone health. Studies have observed an increased abundance of genera *Lactobacillus*, *Actinomyces*, *Eggerthella* in elderly osteopenia and osteoporosis cases, in both men and women^(^ ^26^ ^)^; negative associations of the bacterial order Clostridiales and family *Lachnospiraceae* with heel BMD^(^ ^27^ ^)^; and increased abundance of the genus *Bacteroides* in osteoporotic and osteopenic postmenopausal women.^(^ ^28^ ^)^

In this study, we investigated whether PPI‐induced changes in the gut and/or host metabolic environments may be involved in the modulation of BMD, using phenotypic, gut microbiome, and fecal and plasma metabolomics data from more than 5000 individuals belonging to the well‐characterized TwinsUK cohort.^(^ ^29^ ^)^

## Subjects and Methods

### The TwinsUK cohort

The TwinsUK cohort consists of more than 14,000 volunteers, mostly middle‐aged females, who have participated over the last 29 years in a longitudinal cohort study. This has included lifestyle and health questionnaires, biomedical measurements, biological sample collection, and the generation of multi‐omics profiles (such as genetics, metagenomics, and metabolomics), over multiple visits.^(^ ^29^ ^)^

### Clinical data

BMD was measured at multiple sites by dual‐energy X‐ray absorptiometry (DXA) using standard protocols, as previously described.^(^ ^30^ ^)^ Lumbar spine (LS, L_1_–L_4_), femoral neck (FN), and total hip (TH) BMD measurements were used in this study. DXA was also used to measure total body fat mass,^(^ ^29^ ^)^ which was normalized by dividing it by height squared.^(^ ^31^ ^)^

Frailty index (FI), capturing the overall health status of elderly individuals, was calculated using the Rockwood method, which included 36 domains of potential health deficits based on clinical measurements and self‐reported questionnaire answers.^(^ ^32^ ^)^

### Medication data

Self‐reported use of prescribed medications was available from three questionnaires, collected between 2004 and 2015 from 7873 individuals. Although all questionnaires allowed reporting of medication using a free‐text input, one questionnaire also included selection from a list of drug classes, including, antacids and drugs for stomach ulcer/gastritis, with the option of reporting the exact drug name.

To process free‐text inputs, we first purged extra words (eg, ‘tablets’, ‘MG’, ‘per day’), and stripped white spaces. To extract drug name, substance, and class, we used a fuzzy string match method, ie, the optimal string alignment (osa; implemented in the R package ‘stringdist’, v. 0.9.6.3^(^ ^33^ ^)^), which calculates the distance between two strings, allowing different penalty weight for deletion, insertion, substitution, and transposition (weight assigned were 0.6, 0.6, 0.75, 0.9, respectively). The osa method was used to match our preprocessed free‐text input with records in British National Formulary (BNF, version 01‐01‐2019:76) and select those with the smallest distance, while discarding matches with a distance >2. For the questionnaire suggesting drug classes, to discard over‐the‐counter medications, we used the osa method to revalidate the drugs based on their name, when available. If the drug name was not available, the information was discarded. All records were rechecked manually (XZ, DW, RY, MT) and validated by a clinician (HD).

In total, 7738 individuals were retained. Individuals who never reported using PPI were considered nonusers, while those with at least one record of PPI use were considered as users. The date when they reported being a user or nonuser was recorded. For all association analyses with PPI, we used only data (ie, metagenomics, metabolomics, and BMD) whose collection dates were later than the recorded PPI usage date.

### Metagenomics data

Whole metagenome shotgun sequencing was available for 1004 samples. Details on sample collection, DNA extraction, library preparation, sequencing, and quality control were described previously.^(^ ^34^ ^)^ We used the MetaPhlAn (v 3.0) and HUMAnN (v 3.0) pipelines^(^ ^35^ ^)^ to characterize the composition and functional capability of the microbial community. From the HUMAnN output, we discarded stratified results for MetaCyc microbial metabolic pathways and gene annotations (ie, pathway and gene counts assigned to specific species), and used only the total counts observed in the whole microbial community. UniProt (https://www.uniprot.org) was used to retrieve information about gene families, the encoded protein, and the lineage and taxonomy they belonged to, based on accession numbers provided by HUMAnN. The abundances were normalized with respect to the total number of reads. Microbial taxa were expressed as percentages, whereas microbial metabolic pathways and genes were expressed as copies per million. We converted nil abundances to missing values, because it is uncertain whether these meant a real absence of a taxon, pathway or gene, or the inability to detect it because of technical/sampling issues.^(^ ^34^ ^)^ Data points that were four standard deviations away from the group mean were filtered out as outliers. The relative abundances of taxa were arcsine square‐root transformed. Relative abundances of microbial metabolic pathways and genes were inverse‐normal transformed.

### Metabolomics data

Fecal metabolomics was measured in 786 stool samples, with a semi‐untargeted UPLC–MS/MS platform by Metabolon, Inc., Morrisville, NC, USA, as described.^(^ ^36^ ^)^ After annotation, 266 of 1116 metabolites had unknown chemical identities and were removed from the analyses. Semi‐untargeted plasma metabolomics was measured on 5003 samples from fasting individuals using the UPLC–MS/MS platform by Metabolon, Inc., Morrisville, NC, USA, as described.^(^ ^30^ ^)^ After annotations, 228 of 512 metabolites had unknown chemical identities and were removed from the analyses. Both fecal and plasma metabolites were scaled by run day median; zero values were converted to missing, and the log‐transformed data were standardized to have mean 0 and standard deviation 1.

### Statistical analysis

Differences in age, FI, body mass index (BMI), and height‐adjusted body fat mass between PPI users and nonusers were assessed with the Wilcoxon rank‐sum test, whereas the difference in sex distribution was assessed with the χ^2^ test (R “stats” package, v 3.6.2).

We tested the association between PPI use and BMD (at LS, FN, and TH) using a linear mixed model (‘lmerTest’ R package; v 3.1–3), with family structure included as a random effect to correct for the non‐independence of twin observations. The typical way to accommodate family data is to use linear mixed models where the covariance of the analyzed traits between relatives is modeled based on their degree of relationship, or kinship, as a random effect. Drugs used to treat osteoporosis (ie, bisphosphonates, menopausal hormone therapy [MHT], vitamin D and calcium supplements [only when prescribed by doctors]),^(^ ^37^, ^38^, ^39^, ^40^ ^)^ were associated with BMD, and (collectively) included as covariates together with age, sex, and BMI. Corticosteroid use was not associated with BMD of any region (acknowledging the small number of individuals using corticosteroids (*n* = 120) within our population overall) and therefore not considered further in our analyses. Drugs and supplements were coded as 0 = not taking the drug/supplement or 1 = taking the drug/supplement. FI was not included as a covariate in the BMD association analyses because its calculation included multiple information on bone fractures and doctor‐diagnosed osteoporosis. Association was considered significant if its *p* value was lower than a conservative Bonferroni‐derived threshold of 0.05/3 = 0.016.

We tested for association between PPI and microbial taxa, metabolic pathways, gene families, and fecal and plasma metabolites, using the model described above and including age, sex, BMI, and FI as fixed effects and family structure as random effect. Only taxa, pathways, and genes with at least 30 observations were included in the analyses. Associations passing a false discovery rate (FDR) of 5% were considered significant. FDR was evaluated using Storey's method^(^ ^41^ ^)^ (“qvalue” R package; v 2.18.0). FDR for microbial taxa was evaluated independently at the different taxonomic levels. The percentage each taxon contributed to the microbial genes associations was calculated by dividing the number of genes assigned to that taxon by the number of genes with available taxonomy information. In addition to individual species and genus, we aimed to assess the gut microbial community functions as a whole, summarized as microbial pathways and microbial genes present in the community. Indeed, microbial pathways and genes assigned to specific taxa would be mostly proportional to the abundance of the taxa itself. On the other hand, genes and pathways in the community are representative of the potential of the community itself to carry out a particular metabolic process, and they mainly belong to unknown or unassignable species.

We tested for association between BMD and microbial taxa, metabolic pathways, genes, and plasma metabolites that were significantly associated with PPI use using the model described above and including the use of drugs/supplements used to treat osteoporosis as additional covariate. Associations passing a conservative Bonferroni‐derived threshold of 0.05/*N*, where *N* is the number of tested features, were considered significant. Sensitivity analyses to confirm significant associations with plasma metabolites were carried out in a subset of postmenopausal women who were not using MHT, including age, bisphosphonates, vitamin D and calcium use, and height‐adjusted body fat mass as covariates. An association was considered as significant when its *p* value was lower than a conservative Bonferroni‐derived threshold of 0.05/*N*. Pairwise correlations between metabolites were calculated using Pearson's correlation (R ‘stats’ package, v 3.6.2).

We carried out an enrichment analysis to identify overrepresented super‐pathways using the annotation provided by Metabolon and the PAGE algorithm (as implemented in the R package ‘piano’, v 2.2.0). Signed *t* values from the association analysis were used as input, and multiple testing correction was performed using the ‘fdr’ adjustment. Enrichments with an adjusted *p* value <0.05 were considered significant.

The mediation analyses were carried out using the R package ‘mediation’ (v 4.5.0, function ‘mediate’) with default settings. Use of PPI, metabolite levels, and BMD were fitted into the model as predictor, mediator, and response variable, respectively. Age, sex, BMI, and use of drugs used to treat osteoporosis were included as covariates. We compared two models: (1) PPI use directly influences TH BMD level, and (2) PPI use regulates the metabolite level and indirectly influences the TH BMD. Tests showing a *p* value lower than a Bonferroni‐derived threshold of 0.05/*N* were considered significant.

**Fig. 1:**
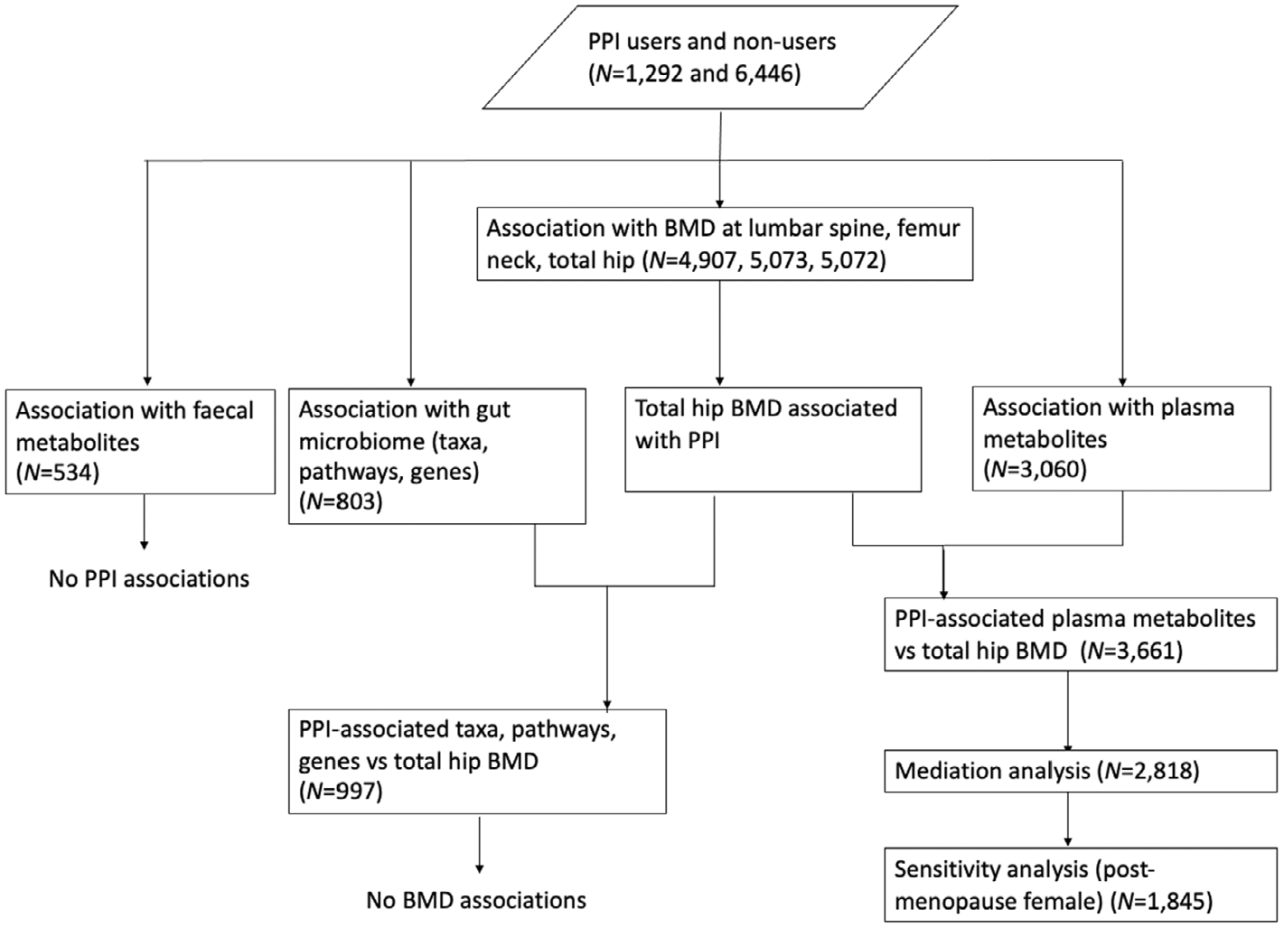
Flowchart of the study design. We retained 7738 individuals who reported clear answer on PPI use from questionnaires, collected during 2004 to 2015. We tested for association between PPI and microbial taxa, metabolic pathways, gene families, and fecal and plasma metabolites, using mixed model and including age, sex, BMI, and FI (for microbial features) as fixed effects, family structure as random effect. We also tested for the association between PPI and BMD at FS, FN, TH sites, using the same mixed model but included the use of osteoporosis drugs and did not include FI. No fecal metabolites were associated with PPI. Next, we tested for association between TH‐BMD (strongest association with PPI) and microbial taxa, metabolic pathways, genes, and plasma metabolites that were significantly associated with PPI use. Only plasma metabolites showed associations with both BMD and PPI, and they were further tested with mediation analysis. Sensitivity analysis was carried out in postmenopausal female individuals, to further confirm the association of these metabolites with PPI and BMD. BMD = bone mineral density; FN = femoral neck; LS = lumbar spine (L_1_–L_4_); PPI = proton pump inhibitor; TH = total hip.

A flowchart of the study design is presented in Fig. 1.

## Results

### Characteristics of PPI users

A total of 1292 individuals from the TwinsUK cohort reported using PPI in at least one of the questionnaires (users), while 6446 reported not using it at any time point (nonusers, Subjects and Methods).

Age, BMI, and height‐adjusted body fat mass were higher in PPI users than nonusers (*p* < 4.0 × 10^−57^; Table 1), suggesting differential underlying health status of users versus nonusers. PPI users also had a higher FI than nonusers (Wilcoxon rank‐sum test, *p* = 3.2 × 10^−65^; Table 1).

**Table 1:** Participant Characteristics

Characteristic	All	Users	Non‐users	*p*
*N*	7738	1292	6446	–
Sex, *n* (%)				4.0 × 10^−3^
Female	4972 (87.8)	1086 (90.5)	5101 (98.5)	
Male	692 (12.2)	114 (9.5)	77 (1.5)	
Age (years), mean ± SD	51.88 ± 15.14	62.52 ± 11.55	49.71 ± 14.86	6.4 × 10^−168^
BMI (kg/m^2^), mean ± SD	26.02 ± 5.03	28.04 ± 5.27	25.61 ± 4.87	4.0 × 10^−57^
Frailty index, mean ± SD^a^	0.20 ± 0.10	0.25 ± 0.11	0.19 ± 0.09	3.2 × 10^−65^
Height‐adjusted fat mass, mean ± SD^b^	9.85 ± 3.69	11.43 ± 3.70	9.53 ± 3.60	3.4 × 10^−57^

Continuous values are reported as mean ± standard deviation (SD) and compared using the Wilcoxon rank‐sum test. Categorical values are reported as number (percentage) and compared using the χ^2^ test.

^a^ Frailty index was available for 6216 individuals with PPI use recorded (*N*_users_ = 1137, *N*_nonusers_ = 5079).

^b^ Height‐adjusted fat mass was available for 6195 individuals with PPI use recorded (*N*_users_ = 1072, *N*_nonusers_ = 5123).

### BMD is decreased in PPI users

We considered BMD at LS, FN, and TH, and a total of 5073, 5072 and 4907 individuals were included in our analyses for the three sites, respectively. For PPI users, only BMD measurements from individuals taking PPI before the DXA scan date were considered. The mean difference between the scan date and the first reported PPI use was 3.50 years (SD = 3.48 years).

For all sites, we tested the difference in BMD between user and nonuser groups, correcting for age, sex, BMI, and use of bisphosphonates, MHT, vitamin D, and calcium supplements (Subjects and Methods). BMD at TH was negatively associated with PPI use (*p* < 0.05/3 = 0.016; Table 2). Not all subjects continued to use PPI up to the date of BMD assessment. Therefore, we carried out a sensitivity analysis to assess the effect of PPI cessation on our results. Among 694 PPI users, 99 declared to have stopped using PPI on average 1 year before the DXA scanning, and another 35 gave an unclear answer. When the association analyses were repeated after removing these 134 subjects, TH BMD was still significantly associated with PPI use (*p* = 7.69 × 10^−3^). Besides, TH BMD was not associated with either the FI (which measures multiple health issues) or the number of medications (*p* > 0.05). However, considering polypharmacy, PPI users used more medications (mean = 4.49, SD = 3.41) than nonusers (mean = 1.41, SD =1.86), Wilcoxon test, *p* < 2.2 *×* 10^−16^. To check whether the unequal number of medications was confounding our association, we matched PPI users and nonusers for the number of drugs used, as well as age, sex, and BMI (1:2 case:control ratio). Despite reducing the sample size from 5072 to 1962 individuals (654 users, 1962 nonusers), TH BMD was still significantly associated with PPI use (*p* = 4.85 × 10^−3^).

**Table 2:** Associations Between PPI Use and Bone Mineral Density

Site	*N*_all_	Mean ± SD (g/cm^2^)	*N*_users_	Mean ± SD (g/cm^2^)	*N*_non‐users_	Mean ± SD (g/cm^2^)	Beta	SE	*p*
Lumbar spine	4907	*1.09 ± 0.19*	670	1.08 *±* 0.19	4,237	1.09 *±* 0.19	0.006	0.007	0.389
Femur neck	5073	0.77 ± 0.13	695	0.74 ± 0.12	4,378	0.78 ± 0.13	−0.009	0.004	0.030
Total hip	*5072*	*0.92 ± 0.14*	*694*	*0.89 ± 0.13*	*4,378*	*0.93 ± 0.14*	*−0.012*	*0.004*	*2.35 × 10* ^*−3*^

For each site, the number of individuals used in the association study (*N*) as well as of PPI users (*N*_users_) and non‐users (*N*_nonusers_), and BMD means and standard deviations (SDs) in each group. Association results are reported as effect size (Beta), standard error (SE), and *p* value. Significant results (Bonferroni‐corrected threshold, *p* < 0.05/3 = 0.016) are in italics.

### The gut microbiome is associated with PPI use but not with BMD

A total of 120 PPI users and 683 nonusers had fecal samples collected after reporting PPI use, and had available FI. The mean difference between the metagenomics assessment and the first reported PPI use was 6.91 years (SD = 1.80 years).

In total, 49 families, 112 genera, and 281 species present in at least 30 samples were tested for association with PPI use (Subjects and Methods). Two families, three genera, and five species were associated with the use of PPI (FDR <5%, Table S1). We identified positive associations with the families *Streptococcaceae* and *Micrococcaceae*, and the genera *Streptococcus* and *Rothia*; and a novel negative association with the genus *Gemmiger*. At the species level, *Streptococcus salivarius*, *Streptococcus parasanguinis*, *Rothia mucilaginosa*, and *Eubacterium* sp. *OM08‐24* showed positive association with PPI use, while *Gemmiger formicilis* was negatively associated. A small number of subjects (*n* = 22) were using antibiotics prior to fecal sample collection, but adding antibiotic use as a covariate had a negligible effect on the association results (Table S2).

Next, we tested for association between 551 Metacyc microbial metabolic pathways and PPI use (Subjects and Methods), identifying 152 (28%) significant associations (FDR <5%; Table S3). These metabolic pathways cover a wide range of microbial functions. Some of the most strongly associated pathways (*p* < 2.5 × 10^−5^) were the superpathway of tetrahydrofolate biosynthesis, polyisoprenoid biosynthesis, and gamma;glutamyl cycle (positively associated) and the transfer RNA (tRNA) charging, peptidoglycan biosynthesis I, and folate transformations II (negatively associated).

Then, we sought association between PPI use and microbial genes (Subjects and Methods). Of approximately 1.57 million microbial gene families tested, 2486 (0.16%) were significantly associated with the use of PPI (FDR <5%, Table S4). After mapping to the UniProt database (Subjects and Methods), 2227 gene records were assigned their protein product and to the specific species they belonged to. Only a small proportion of the encoded proteins (*N* = 375, 16.8%) were uncharacterized, and the remainder were involved in a variety of metabolic pathways essential for maintaining microbial activities, such as membrane protein, histidine kinase, and acetyltransferase. When looking at the taxonomy of each gene family (Table S5), we noticed that the PPI‐associated genes belonged to taxa (families, genera, or species) that were also associated with PPI in the analysis presented above, further validating these associations. For instance, half of the associated genes belonged to bacteria belonging to the family *Streptococcaceae* (50%) and/or the genus *Streptococcus* (50%), and, consequently, to a large proportion of the species *S. parasanguinis* (14%) and *S. salivarius* (13%). Analogously, PPI‐associated genes belonged to the genus *Gemminger* (15%), including the species *G. formicilis* (15%), and *Subdoligranulum* (15%).

Next, we investigated whether there was an association between 850 known fecal metabolites and PPI use, in a sample of 125 PPI users and 409 nonusers (Subjects and Methods). No association was found after correcting for multiple testing (FDR <5%).

Last, to investigate whether PPI use contributed to the association between BMD and the gut microbiome, we tested whether microbial taxa, metabolic pathways, and gene families associated with PPI use were also associated with BMD at TH (which showed the strongest association with PPI in our analysis). We did not find associations, suggesting that the gut microbiome is not mediating PPI‐induced BMD change.

### PPI‐induced changes in plasma metabolite levels influence total hip BMD

Plasma metabolomics, assessing 284 known metabolites, were available for 3060 individuals whose samples were collected after reporting PPI use (*N*_users_ = 339, *N*_non‐users_ = 2721; Subjects and Methods). The mean difference between the plasma metabolomics assessment and first reporting PPI use was 1.55 years (SD = 1.72 years).

We identified significant associations between PPI use and 40 metabolites (FDR <5%; Subjects and Methods, Table S6). Enrichment analysis showed a decrease of lipids compared to the whole metabolomics dataset (PAGE adjusted *p* = 8 × 10^−4^, Subjects and Methods).

We then tested the association between these 40 plasma metabolites and TH BMD in a subset of 3661 individuals (Subjects and Methods). We found nine lipids associated with TH BMD (*p* < 0.05/40 = 1.3 × 10^−3^; Table S7). Seven were sulfated steroids, all involved in androgen pathways and highly correlated in our sample (Fig. S1) – viz. dehydroisoandrosterone sulfate (DHEA‐S), androsterone sulfate, epiandrosterone sulfate, 5alpha‐androstan‐3beta, 17beta‐diol disulfate, 4‐androsten‐3beta, 17beta‐diol monosulfate, and 4‐androsten‐3beta, 17beta‐diol disulfate (1) * and (2) *. Additionally, 3‐carboxy‐4‐methyl‐5‐propyl‐2‐furanpropanoate (CMPF) and 10‐undecenoate (11:1n1) were associated with BMD but were not correlated with the group of sulfated steroid metabolites (Fig. S1).

We observed associations between the identified nine metabolites and age, sex, height‐adjusted body fat mass, and MHT use (*p* < 0.05; data not shown). Therefore, we carried out a sensitivity analysis in a subset of 1854 postmenopausal women not on MHT, where we tested the association between TH BMD and these nine metabolites, correcting for age, height‐adjusted body fat mass, use of bisphosphonates and/or vitamin D and calcium supplements (Subjects and Methods). All associations were confirmed (Table S8). In our analyses we set the metabolomics values below the detection limit as missing. To further assess the robustness of our results further, we re‐ran the analyses with values under detection imputed with runday minimum, obtaining analogous results (Table S9).

**Fig. 2:**
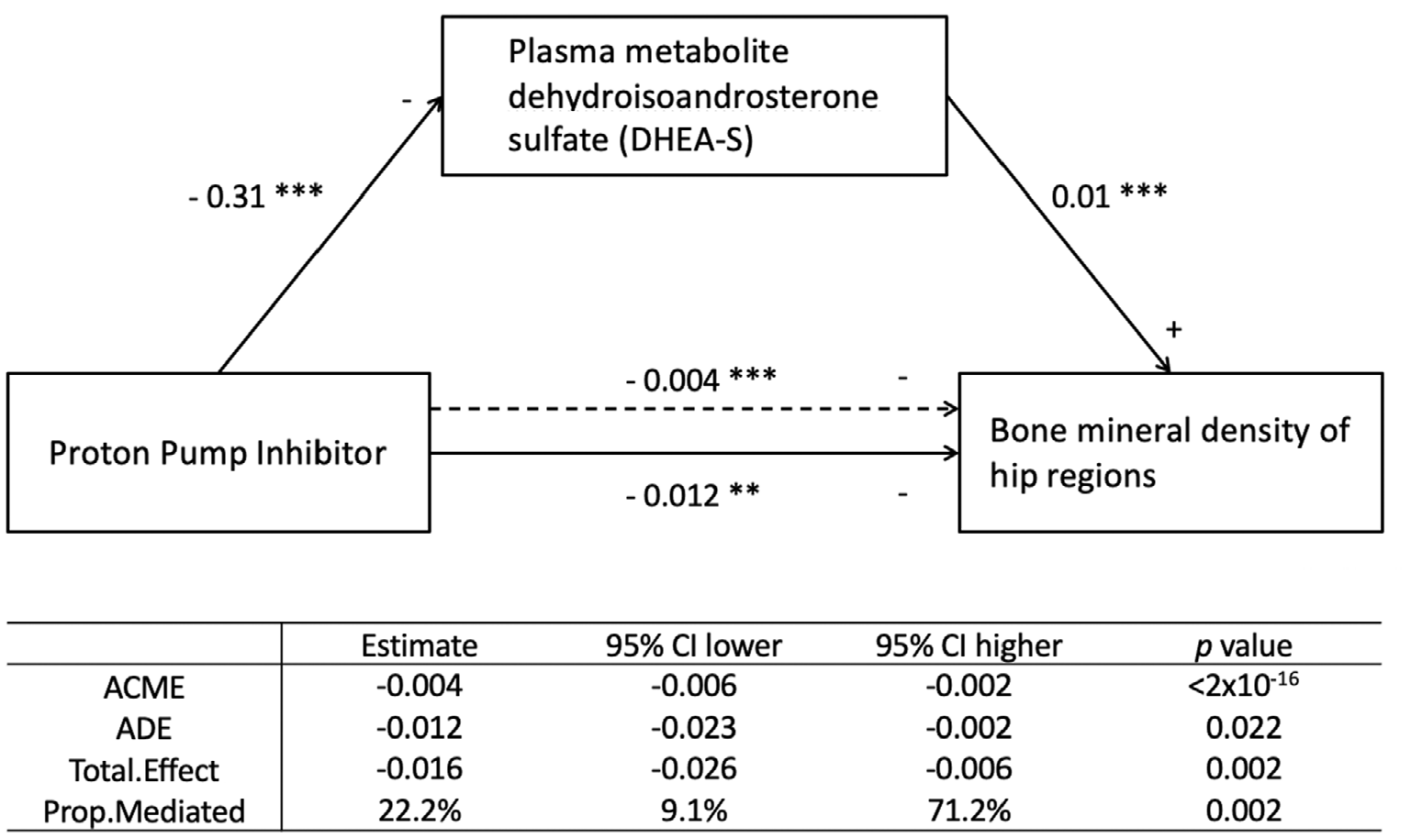
Direct effect and indirect mediation effect via the plasma metabolite DHEA‐S of PPI use affecting total hip BMD. The solid lines show direct associations. The dotted line shows the total indirect effect. For each association, the regression coefficient of the linear mixed‐effects model is reported along its significance level (**p < 0.05, ***p < 0.001). The total effect is the sum of ACME and ADE, and “prop.mediated” shows the percentage of effect that went through the mediator. ACME = average causal mediation effect; ADE = average direct effect; DHEA‐S = dehydroisoandrosterone sulfate.

Because these nine plasma metabolites were associated with both PPI use and TH BMD, we hypothesized that they may modulate PPI's effect on BMD. We tested this hypothesis using mediation analysis, which tests how one variable (PPI, in our case) influences another (TH BMD) through a mediator (a plasma metabolite; Subjects and Methods). Age, sex, BMI, and use of drugs used to treat osteoporosis were included as covariates. All nine metabolites showed potential mediation effect (*p* < 0.05/9 = 5.6 × 10^−3^), with concordant directions of effects (Table S10 and Fig. S2). For example, we observed decreased plasma level of DHEA‐S in PPI users, whereas DHEA‐S level was positively associated with TH BMD, and PPI use was negatively associated with TH BMD (Fig. 2). Of the total effect of PPI use on BMD, 22.2% went through the mediator. Similar results were obtained for the other highly correlated metabolites but the effect through the mediator was lower for the nonsulfated steroids metabolites CMPF, 10%, and 10‐undecenoate (11:1n1, 5%; Table S10).

## Discussion

In this study, we observed significantly lower BMD in TH (*p* = 8.3 × 10^−4^) and FN (*p* = 0.015) in PPI users. Further, we identified two families, three genera, and five species associated with PPI use, confirming an increase of pharyngeal‐driven bacterial families of *Micrococcaceae* and *Streptococcacea* in the gut of PPI users.^(^ ^19^, ^20^, ^42^ ^)^

Five species showed significant positive association, including the oral‐driven *S. salivarius*, *S. parasanguinis*, and *R. mucilaginosa*, and the gut commensal *Eubacterium* sp. *OM08‐24*, while *G. formicilis* was negatively associated. And the association with *Eubacterium* sp. *OM08‐24* and *G. formicilis* were novel. *G. formicilis* is a very common species in our dataset, found in 92% of the analyzed samples. It has been previously associated with Ipilimumab‐induced colitis in patients with melanoma^(^ ^43^ ^)^; with dextran sulfate sodium‐induced colitis in mice^(^ ^44^ ^)^; and with the presence of blood in feces in colorectal cancer patients.^(^ ^45^ ^)^ Conversely, *Eubacterium* sp. *OM08‐24* and unclassified *Eubacterium* are less common in our dataset, found in 13% of our samples.

Additionally, 152 microbial metabolic pathways were significantly associated with PPI use, suggesting a wider effect of PPI use on gut microbial metabolism. We replicated 53% (88/166) of the pathways identified by Vila and colleagues,^(^ ^21^ ^)^ using a different version of HUMAnN (v 0.10.0 versus v3.0). The newly associated pathways include super pathways of menaquinol biosynthesis, tetrahydrofolate biosynthesis, and heme biosynthesis. *E. coli* was the major contributor to these pathways, and the association is likely to be explained by differences in *E. coli* abundances in PPI users and nonusers.

PPI use was also significantly associated with about 2500 microbial genes. These genes cover a wide range of microbial functions, and when we mapped the genes to microbial species using the UniProt database, about half of the genes belonged to the *Streptococcus* family, mostly *S. parasanguinis* and *S. salivaris*, the two species most strongly associated with PPI use, but also to *G. formicilis*, thus further supporting our findings. Despite PPI use showing significant effects on the gut microbial composition and functions, we observed no association between BMD and the gut microbiome in terms of taxa, microbial metabolic pathway, and genes that associated with PPI in our sample, thus suggesting that the gut microbiome is unlikely mediating PPI effects on BMD.

On the other hand, we identified significant associations between PPI use and 40 plasma metabolites. Interestingly, nine plasma metabolites significantly decreased in PPI users were also associated with lower TH BMD: 3‐carboxy‐4‐methyl‐5‐propyl‐2‐furanpropanoate (CMPF), 10‐undecenoate (11:1n1), and seven androgen sulfates strongly correlated with each other, such as DHEA‐S, androsterone sulfate and 4‐androsten‐3beta, 17beta‐diol disulfate (1) * and (2) *.

Androgen levels can increase as women transition through menopause, with loss of ovarian function but maintained adrenal function, and altered sex hormone‐binding globulin levels (SHBG).^(^ ^46^, ^47^ ^)^ BMI, body fat mass, and body fat percentage are associated with plasma DHEA/DHEA‐S levels, with most studies observing negative associations between DHEA and body fat, while less consistent results were observed for DHEA‐S.^(^ ^48^ ^)^ Menopausal hormone therapy provides an exogenous source of estrogen that can also influence sex hormone levels, through effects on SHBG levels. Therefore, we carried out a sensitivity analysis using a selected subset of postmenopause women not using MHT, and adjusting for age, use of bisphosphonates and/or vitamin D and calcium supplements as well as height‐adjusted body fat mass, which confirmed the associations between these nine metabolites and TH BMD. Finally, analyses showed significant mediation of PPI effect on TH BMD directly or indirectly via the regulation of these metabolites, with DHEA‐S having the largest mediation proportion (22.2%). These results suggest a potential mechanism for PPI influence on BMD, although we cannot rule out the possibility that such effect may be achieved via changes in calcium absorption.

DHEA‐S, secreted by the adrenal glands, is the sulfonated form of dehydroepiandrosterone (or dehydroisoandrosterone, DHEA). Although circulating at much high concentrations relative to other steroid sex hormones (including DHEA) DHEA‐S is relatively inert and does not bind to normal androgen receptors.^(^ ^49^ ^)^ DHEA‐S can be converted back to DHEA, which is a precursor for the synthesis of more potent androgens (ie, testosterone and DHT), and, through their aromatization, estrogens. Both estrogens and androgens are key regulators of bone metabolism.^(^ ^50^ ^)^ DHEA‐S levels have been suggested to predict LS BMD in older men^(^ ^51^ ^)^ and high DHEA‐S level was predictive of decreased bone loss at LS and FN in women, after adjusting for baseline estradiol and hormone replacement therapy.^(^ ^52^ ^)^ A recent study using 2‐sample Mendelian randomization suggested a causal role of high DHEA‐S on LS BMD increase, with genetically predicted higher DHEA‐S levels associating with decreased forearm fracture risk in women, showing the first causal link between DHEA‐S and fractures.^(^ ^53^ ^)^ However, no other studies have shown that DHEA or DHEA‐S have an effect on fracture; and the extent to which any observed effects on BMD are mediated through metabolism to much more potent sex hormones is unclear. Importantly, estrogen, testosterone, and many other potent sex steroids were not measured on our metabolomics platform, limiting our ability to attribute our findings definitively to DHEA‐S per se rather than any downstream metabolite(s).

Other BMD‐associated and PPI‐associated plasma metabolites showed high correlation with DHEA‐S, such as 4‐androsten‐3beta, 17beta‐diol disulfate (1) * and (2) *, 4‐androsten‐3beta, 17beta‐diol monosulfate and 5alpha‐androstan‐3beta, 17beta‐diol disulfate. These are 19‐carbon steroids with one or two sulfate groups attached to the steroid skeleton. The hydroxyl form of these steroid sulfates belongs to steroid hormone biosynthesis or degradation pathways. CMPF is a furoic acid and a potent uremic toxin. Decreased CMPF levels were associated with lower BMD in a recent small study in 69 Arabic individuals,^(^ ^54^ ^)^ and in 701 postmenopausal Chinese women.^(^ ^55^ ^)^ 10‐undecenoate (11:1n1) is a medium‐chain fatty acid, associated with excessive alcohol^(^ ^56^ ^)^ and butter consumption.^(^ ^57^, ^58^ ^)^

The TwinsUK cohort is an ideal sample to identify generalizable mechanisms of bone loss, because volunteers are representative of the general population. Moreover, the availability of multiomics data allows observation and comparison of many different facets of the physiological state. Thus, although our sample size is reasonably large (*N* = 4907, 5073, 5072 for LS, FN, TH regions, respectively) but not the largest in the literature—and medication data are self‐reported—we believe that our observation on the potential involvement of the androgen pathways in bone loss deserve additional investigations.

Despite the strength of our study due to the integration of multiomics data in the well‐characterized TwinsUK cohort, there are also a number of limitations to acknowledge. First, other factors not included in this study may influence the level of plasma metabolites in our analyses; eg, differing food intakes. For instance, CMPF was identified as a prominent metabolite after fish oil and fish consumption,^(^ ^59^ ^)^ and serum 4‐androsten‐3beta, 17beta‐diol disulfate and 10‐undecenoate (11:1n1) were associated with alcohol intake^(^ ^56^, ^60^ ^)^ and butter consumption.^(^ ^57^, ^58^ ^)^ Second, we assumed continuous use of PPIs for all PPI users included in these analyses. However, some individuals may have interrupted their PPI use during our study period, which might be missed by our questionnaires which are sent out at regular intervals every 3 to 4 years. Third, many other drugs are co‐prescribed with PPIs. To mitigate this issue, we included drugs or supplements used to decrease BMD loss in our analyses. Last, we acknowledge our limited number of subjects with microbiome data, especially compared to the number of subjects with metabolomics data, which may have reduced our ability to detect microbial associations with BMD. In this context, a recent study from Dekkers and colleagues in 2022^(^ ^61^ ^)^ identified association between gut microbes part of the normal oral microbiota (including *S. salivarius* and *S. parasanguinis*) and both PPI use and androgen sulfates metabolites. However, this study did not combine PPI use and microbial abundance data in the metabolomics association tests, thus leaving open the question of whether androgen sulfates levels were mainly influenced by PPI use or by PPI‐associated microbes.

In summary, we observed a decrease in LS and TH BMD in PPI users. We also confirmed multiple associations between PPI use and gut microbial taxa, microbial metabolic pathways, and genes, and two novel associations with species, although no association was observed between these and BMD, suggesting that the gut microbiome is not playing a role in PPI's effect on BMD. However, our investigation of the systemic host metabolism highlighted that PPI use may influence TH BMD both directly and indirectly mainly via the regulation of plasma metabolites involved in the sex hormone pathway.

## Conflict of Interest

TDS is a founding shareholder and consultant for ZOE Ltd. CJS is a consultant for ZOE Ltd. The other authors have nothing to disclose.

## Author Contributions

**Xinyuan Zhang:** Writing ‐ original draft; Writing ‐ review & editing; Methodology; Investigation; Formal analysis; Visualization; Conceptualization; Data curation. **Adewale S. Adebayo:** Data curation. **Dongmeng Wang:** Data curation. **Yasrab Raza:** Data curation. **Max Tomlinson:** Data curation. **Hannah Dooley:** Data curation. **Ruth C.E. Bowyer:** Data curation. **Kerrin S. Small:** Data curation; Supervision. **Claire J. Steves:** Data curation; Supervision. **Tim D. Spector:** Supervision; Funding acquisition. **Emma L. Duncan:** Writing ‐ review & editing; Supervision; Methodology. **Alessia Visconti:** Methodology; Data curation; Supervision; Writing ‐ review & editing; Conceptualization. **Mario Falchi:** Writing ‐ review & editing; Supervision; Methodology; Conceptualization.

### Peer Review

The peer review history for this article is available at https://publons.com/publon/10.1002/jbmr.4754.

## Supporting information


**Fig. S1.** Correlations of plasma metabolites that are associated with both PPI and hip BMD.
**Fig. S2.** Direct effect and indirect mediation effect via plasma metabolites of PPI use affecting total hip BMD.

**Table S1.** Microbial taxa associated with PPI.
**Table S2.** Microbial metabolic pathways associated with PPI.
**Table S3.** Microbial gene families associated with PPI.
**Table S4.** Taxonomy information for the genes associated with PPI.
**Table S5.** Plasma metabolites associated with PPI.
**Table S6.** Plasma metabolites associated with PPI use and hip BMD.
**Table S7.** Sensitivity analyses of plasma metabolites associated with PPI use and hip BMD.
**Table S8.** Results for the mediation analysis.
**Table S9.** Sensitivity analysis showing the association results for microbial taxa associated with PPI after adding antibiotic use as a covariate.
**Table S10.** Plasma metabolites associated with PPI use and hip BMD using runday minimum imputed data.

## Data Availability

The original contributions presented in the study are included in the article/Supplementary Material. Raw data on TwinsUK participants are available to bona fide researchers under managed access due to governance and ethical constraints. Raw data should be requested via the TwinsUK website (http://twinsuk.ac.uk/resources-for-researchers/access-our-data/) and requests are reviewed by the TwinsUK Resource Executive Committee (TREC) regularly.
