# Supplementary material for: PPI‐Induced Changes in Plasma Metabolite Levels Influence Total Hip Bone Mineral Density in a UK Cohort
Source: J Bone Miner Res. 2022 Dec 30;38(2):326–34. doi: 10.1002/jbmr.4754 (PMC10108201; doi:10.1002/jbmr.4754)
Supplement: Supplementary file 1 — Fig. S1. Correlations of plasma metabolites that are associated with both PPI and hip BMD. Fig. S2. Direct effect and indirect mediation effect via plasma metabolites of PPI use affecting total hip BMD. [file JBMR-38-326-s002.pdf]

Supplementary Figures and Tables for:

PPI-induced changes in plasma metabolite levels influence total hip bone mineral density in a UK cohort

Zhang *et al.*

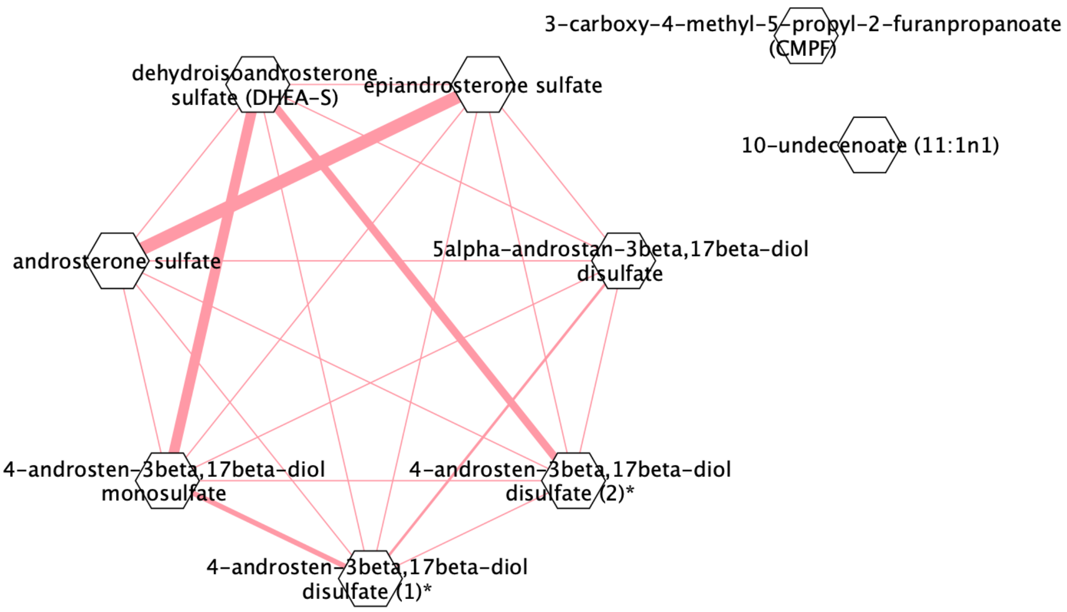

Supplementary Figure 1. Correlations of plasma metabolites that are associated with both PPI and hip BMD. Pairwise Pearson's correlation tests were performed. Each node represents a metabolite, while edges show the Pearson's correlation. The thickest the line, the strongest the correlation. Only the Pearson's correlations larger than 0.5 are shown. The figure was created with the ExpressionCorrelation app (version 1.1.0) in Cytoscape (version 3.9.1).

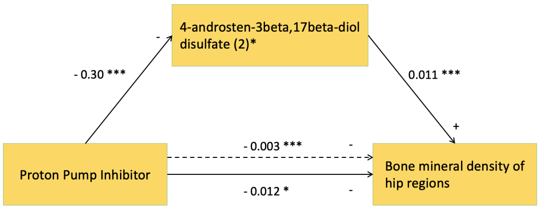

	Estimate	95% CI lower	95% CI higher	*p* value
ACME	-0.003	-0.006	-0.001	$<2x10^{-16}$
ADE	-0.012	-0.024	-0.001	0.034
Total.Effect	-0.016	-0.027	-0.004	0.002
Prop.Mediated	21.4%	6.8%	77.9%	0.002

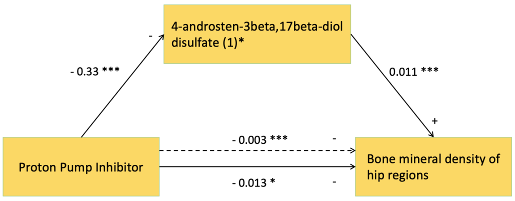

	Estimate	95% CI lower	95% CI higher	*p* value
ACME	-0.003	-0.005	-0.002	$<2x10^{-16}$
ADE	-0.013	-0.024	0.000	0.04
Total.Effect	-0.016	-0.027	-0.005	0.01
Prop.Mediated	21.0%	9.4%	70.9%	0.01

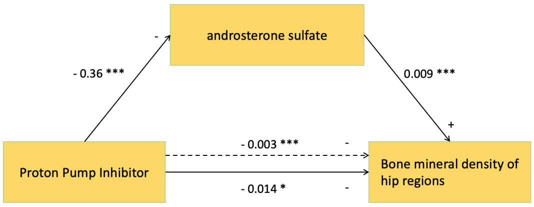

	Estimate	95% CI lower	95% CI higher	*p* value
ACME	-0.003	-0.005	-0.002	$<2x10^{-16}$
ADE	-0.014	-0.024	-0.003	0.016
Total.Effect	-0.017	-0.027	-0.006	$<2x10^{-16}$
Prop.Mediated	19.2%	8.4%	59.4%	$<2x10^{-16}$

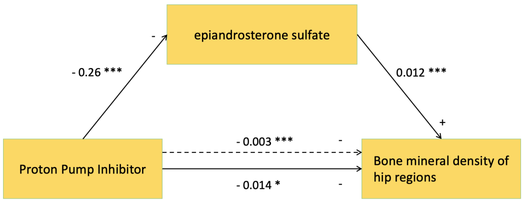

	Estimate	95% CI lower	95% CI higher	*p* value
ACME	-0.003	-0.005	-0.002	$<2x10^{-16}$
ADE	-0.014	-0.025	-0.002	0.014
Total.Effect	-0.017	-0.028	-0.006	0.004
Prop.Mediated	19.0%	8.0%	56.2%	0.004

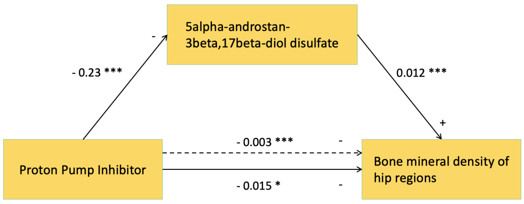

	Estimate	95% CI lower	95% CI higher	*p* value
ACME	-0.003	-0.004	-0.001	$<2x10^{-16}$
ADE	-0.015	-0.027	-0.004	0.012
Total.Effect	-0.018	-0.030	-0.006	0.004
Prop.Mediated	15.4%	6.5%	44.7%	0.004

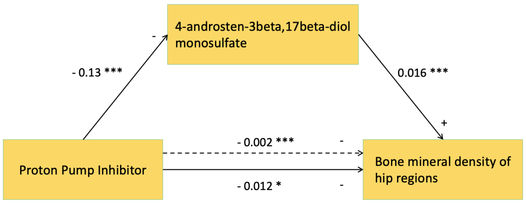

	Estimate	95% CI lower	95% CI higher	*p* value
ACME	-0.002	-0.004	-0.001	$<2x10^{-16}$
ADE	-0.012	-0.023	-0.001	0.036
Total.Effect	-0.014	-0.025	-0.003	0.014
Prop.Mediated	14.1%	3.9%	63.0%	0.014

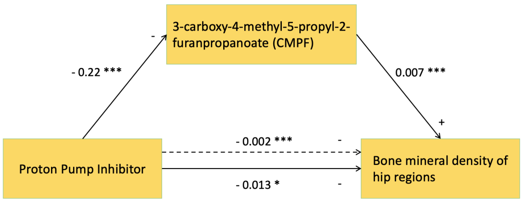

	Estimate	95% CI lower	95% CI higher	*p* value
ACME	-0.002	-0.003	-0.001	$<2x10^{-16}$
ADE	-0.013	-0.024	-0.002	0.024
Total.Effect	-0.015	-0.026	-0.004	0.008
Prop.Mediated	10.0%	3.2%	47.0%	0.008

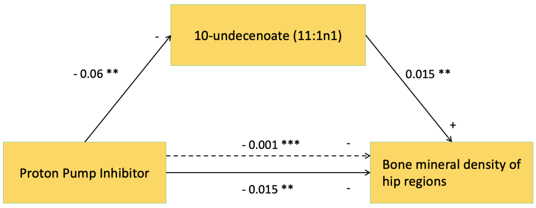

	Estimate	95% CI lower	95% CI higher	*p* value
ACME	-0.001	-0.002	0.000	$<2x10^{-16}$
ADE	-0.015	-0.025	-0.004	0.004
Total.Effect	-0.016	-0.026	-0.005	0.004
Prop.Mediated	5.5%	1.2%	20.7%	0.004

Supplementary Figure 2. Direct effect and indirect mediation effect via plasma metabolites of PPI use affecting total hip BMD. The solid lines show direct associations. In each panel, the dotted line shows the total indirect effect. For each association, the regression coefficient of the linear mixed effects model is reported along its significance level (** $p < 0.05$, *** $p < 0.001$). ACME: average causal mediation effect, ADE: average direct effect, the total effect is the sum of ACME and ADE, and prop.mediated shows the percentage of effect that went through the mediator.
